# ASSOCIATION OF AGE DISCRIMINATION, JOB STRESS, AND DEPRESSION AMONG OLDER WORKERS: ROLE OF SOCIAL NETWORKS

**DOI:** 10.1093/geroni/igz038.467

**Published:** 2019-11-08

**Authors:** Abhijit Visaria, Angelique Chan, Grand Cheng, Rahul Malhotra, Truls Ostbye

**Affiliations:** 1 Duke-NUS Medical School, Singapore, Singapore; 2 Centre for Ageing Research and Education, Duke-NUS Medical School, Singapore, Singapore; 3 Duke-NUS Medical School, Singapore, Singapore, Singapore; 4 Duke University Medical School, Durham, North Carolina, United States

## Abstract

In this study, we examine the association between perceived age discrimination at the workplace and job stress, with depressive symptoms among currently working late-middle-aged adults. In particular, we investigate whether the association between these work-related factors and depression varies by the strength of their social networks. We use data from the Panel on Ageing and Transitions in Health Survey (PATHS) a national study of 1654 Singaporean citizens and permanent residents aged 50 to 59 years, conducted in 2016-2017. We account for age and other sociodemographic characteristics, measures of economic status, occupation, early retirement intentions, personality traits, as well as multiple physical health measures. Our findings indicate that age discrimination at work and job stress are both positively associated with higher depressive symptom scores. The relationship between age discrimination and depressive symptoms however varies by social networks, with age discrimination negatively associated with depressive symptom scores among those with larger friends-based social networks. Our findings indicate that while psychological wellbeing of late middle-aged workers is adversely affected by age discrimination and job stress, these workers benefit from wider and deeper social connections with friends. Our results provide empirical support to previous research that suggests that friends-based social networks yield distinct benefits in terms of subjective well-being and increased self-worth.

